# Combining Pulse Wave Velocity With Galectin-3 to Predict Mortality and Cerebrovascular and Cardiovascular Events in Hemodialysis Patients

**DOI:** 10.3389/fmed.2020.579021

**Published:** 2020-10-20

**Authors:** Qi Zhang, Kanhua Yin, Mingli Zhu, Xinghui Lin, Yan Fang, Jiayue Lu, Zhenyuan Li, Zhaohui Ni

**Affiliations:** ^1^Department of Nephrology, Renji Hospital, School of Medicine, Shanghai Jiaotong University, Shanghai, China; ^2^Division of Nephrology, Shanghai Ninth People's Hospital, School of Medicine, Shanghai Jiaotong University, Shanghai, China; ^3^Harvard T.H. Chan School of Public Health, Boston, MA, United States

**Keywords:** Galectin-3, pulse wave velocity (PWV), cerebrovascular and cardiovascular events, mortality, hemodialysis (HD)

## Abstract

**Background:** Cerebrovascular and cardiovascular diseases contribute substantially to the mortality of end-stage renal disease patients. We sought to combine pulse wave velocity (PWV) with galectin-3 to predict the mortality and cerebrovascular and cardiovascular events in hemodialysis patients.

**Methods and Results:** End-stage renal disease patients who underwent stable hemodialysis were screened for inclusion. Patients with preexisting cardiovascular and cerebrovascular diseases were excluded. The primary endpoint was a composite of all-cause mortality and major adverse cerebrovascular and cardiovascular events. Receiver operating characteristic curve analysis was used to determine the optimal cutoffs to dichotomize PWV and galectin-3. The study population was then stratified into four groups based on these cutoffs. Both univariable and multivariable Cox regression analyses were performed to estimate the hazard ratio and 95% confidence interval (CI) for clinical factors. Model performance was compared among models with or without PWV and galectin-3. A total of 284 patients were enrolled. During a median follow-up of 31 months, 57 patients (20.1%) reached the primary endpoint. The optimal cutoffs for PWV and galectin-3 were 7.9 m/s and 30.5 ng/ml, respectively. In the multivariable regression analysis, the high PWV–high galectin-3 group was associated with a 3-fold increased risk of all-cause mortality and major adverse cerebrovascular and cardiovascular events (hazard ratio = 3.19, 95% CI: 1.05–9.66, *p* = 0.04) compared with the low PWV–low galectin-3 group. The combination of PWV and galectin-3 was associated with improved model discrimination, calibration, and reclassification.

**Conclusions:** The combination of PWV and galectin-3 can be used to predict mortality and cerebral and cardiovascular complications in hemodialysis patients.

## Introduction

Chronic kidney disease (CKD) is a significant clinical and public health problem with a prevalence of up to 15%. The incidence of cerebrovascular and cardiovascular diseases in CKD patients is two times that in those without CKD, and patients with end-stage renal disease (ESRD) are associated with an even higher risk (1). Despite the advances in treatment, cerebrovascular and cardiovascular complications contribute substantially to the mortality of dialysis patients, accounting for 54% of all deaths (2). Early detection of these potentially fatal complications can allow targeted interventions and eventually lead to improved survival and quality of life.

Several non-invasive tests have been developed to predict cerebrovascular or cardiovascular events among hemodialysis patients. Pulse wave velocity (PWV), recommended by the 2018 European Society of Hypertension/European Society of Cardiology hypertension management guidelines as a gold standard for measuring the stiffness of large arteries (3), has been shown to predict mortality and non-fatal cardiovascular events in dialysis patients (4). However, recent studies have revealed that the predictive power of PWV is inferior to that of simple clinical risk scores in ESRD patients (5) and compromised elderly patients (6).

Galectin-3, a member of the β-galactoside-binding lectin family, has emerged as a new prognostic biomarker for a series of cardiovascular diseases, such as congestive heart failure and coronary artery disease (7). It is a 29–35-kDa protein secreted by activated macrophages and other inflammatory cells and plays an essential role in cell adhesion, activation, growth, and differentiation (8). Many research teams, including us, have demonstrated the associations between galectin-3 and multiple cardiovascular complications and survival outcomes in CKD and ESRD patients (9, 10).

Both PWV and galactin-3 measurements are non-invasive tests and can be performed in the dialysis clinic. However, no data have been reported on the predictive power of combining these two assays. In this work, we aimed to combine PWV with galectin-3 measurement to predict the mortality and cerebrovascular and cardiovascular events in hemodialysis patients.

## Methods

### Study Population

ESRD patients who underwent stable hemodialysis at Renji Hospital, Shanghai Jiaotong University, Shanghai, China, between June 2014 and January 2015 were prospectively enrolled. Stable hemodialysis was defined as receiving hemodialysis for three sessions a week, lasting 4 to 5 h each session, for at least 3 months. Patients with the following conditions were excluded: congestive heart failure (New York Heart Association class III or IV or left ventricular ejection fraction <40%), moderate or severe aortic valve stenosis, atrial fibrillation, second- or third-degree atrioventricular block, use of a pacemaker, recent myocardial infarction (MI) (≤3 months), recent stroke (≤3 months), recent transient ischemic attack (≤3 months), pulseless extremity, malignancy, acute infectious diseases (≤3 months), and those who refused to participate in this study.

All enrolled patients signed an informed consent form, and the Ethics Committee of Renji Hospital, Shanghai Jiaotong University, approved this study. This study was performed following the ethical standards in the 1964 Declaration of Helsinki and its later amendments.

### Measurement of Galectin-3, Pulse Wave Velocity, and Blood Pressure

The methods of measuring serum galectin-3, PWV, and blood pressure were described in our previous work (9). In short, we collected patients' blood samples before their midweek dialysis session and measured the serum galectin-3 concentration with an enzyme-linked immunosorbent assay (Human Galectin-3 Quantikine ELISA Kit; R&D Systems Inc., Minneapolis, MN, USA). The baseline laboratory data were collected, including albumin, calcium, phosphorus, creatinine, total triglyceride, total lipoprotein, high-density lipoprotein, low-density lipoprotein, B-type brain natriuretic peptide, and C-reactive protein (CRP). The single-pool Kt/V for urea was used to estimate the dialysis adequacy.

Both the PWV and blood pressure were assessed at the same time as galectin-3. PWV was calculated as the ratio of the distance that the pulse wave traveled (in meters) to the pulse transit time (in seconds) and was assessed using a portable device (Sphygmocor XCEL; AtCor Medical, New South Wales, Australia). The blood pressure was recorded as the average of three consecutive brachial blood pressure readings.

### Primary Endpoint and Definitions of Comorbidities

The primary endpoint of this study was a composite of all-cause mortality and major adverse cerebrovascular and cardiovascular events (MACCE), including acute MI, new-onset heart failure, ischemic stroke, and cerebral hemorrhage. The diagnosis of acute MI required both ECG changes and the elevation of cardiac biomarkers. Heart failure was diagnosed clinically according to typical dyspnea symptoms, pulmonary edema on chest X-ray, and the requirement of additional hemodialysis. Stroke and cerebral hemorrhage were diagnosed by neurologists and confirmed by either magnetic resonance imaging or computed tomography. All enrolled patients were followed until the primary endpoint or July 31, 2017, whichever occurred earlier.

In this study, hypertension was defined as predialysis blood pressure ≥ 140/90 mmHg (11). Diabetes was defined as a fasting plasma glucose level ≥ 7.0 mmol/L, 2-h plasma glucose or random glucose level of ≥11.1 mmol/L with symptoms of hyperglycemia, or an A1C value ≥ 6.5% (12). Prior coronary artery disease was defined as patients with documented significant coronary artery stenosis (>70%) or a history of MI, percutaneous coronary intervention, or coronary artery bypass grafting surgery.

### Risk Stratification, Model Building, Performance Evaluation, and Statistical Analysis

Receiver operating characteristic (ROC) curve analysis was conducted, and the areas under the ROC curve (AUCs) for PWV and galectin-3 were calculated. The optimal cutoffs of PWV and galectin-3 for the classification of the composite endpoint were obtained based on the Youden index's maximization. Based on the ROC-optimal cutoffs for PWV and galectin-3, we further stratified the entire study population into four groups: group 1: low PWV–low galectin-3; group 2: low PWV–high galectin-3; group 3: high PWV–low galectin-3; and group 4: high PWV–high galectin-3. The cumulative incidences of the primary endpoint for these four groups were estimated using the Kaplan–Meier method and compared using the log-rank test.

Univariable Cox proportional-hazards regression was performed to estimate the hazard ratio (HR) and 95% confidence intervals (CIs) of clinical factors, and PWV and galectin-3 were included as continuous and categorical variables (cutoffs derived from the ROC curve analysis were used) separately. A multivariable Cox proportional-hazards model was built with factors associated with the primary outcomes with a two-sided *p* < 0.1 in the univariable analysis combined with other well-known predictive factors. The overall goodness of fit and the proportional-hazards assumption of the Cox regression were assessed based on the Cox–Snell residuals.

We then evaluated the performance of four multivariable Cox models that included different variables and determined whether adding PWV and galectin-3 values would increase the model performance. The model performance was assessed based on discrimination (C statistics), calibration (Hosmer–Lemeshow statistic), and reclassification (net reclassification improvement and integrated discrimination improvement).

The continuous variables were tested for normal distribution by the Kolmogorov–Smirnov test and presented as means ± standard deviations or medians with interquartile ranges as appropriate. Categorical variables were presented as the number and percentage and compared using the chi-square test. For all statistical tests, a *p* < 0.05 was considered significant. All statistical analyses were performed with IBM SPSS 21.0 software (SPSS Inc., Chicago, IL) or the R language statistical software version 4.0.2 (The R Foundation for Statistical Computing, Vienna, Austria).

## Results

### Study Cohort and Baseline Characteristics

**Figure 1:**
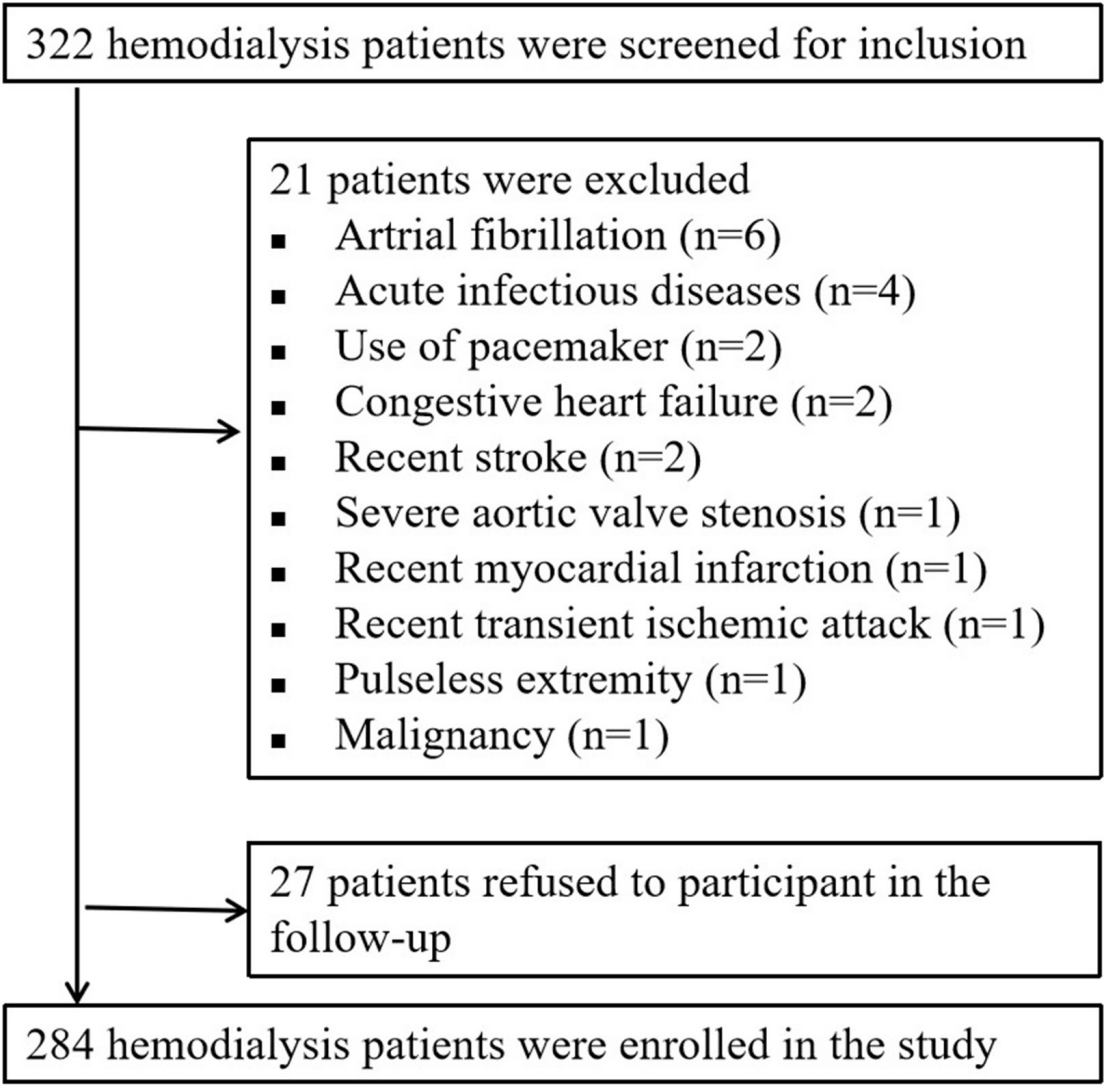
Flow chart of patient enrollment and exclusion.

From June 2014 to January 2015, a total of 332 hemodialysis patients were screened for inclusion. Twenty-one patients were excluded because they met one or more of the exclusion criteria. The other 27 patients were excluded because they refused to participate in the follow-up, resulting in a total of 284 patients eventually included in this analysis (Figure 1).

The median age in this 284-patient cohort was 61 years, and males comprised 58.1% of the cohort. All patients had undergone stable hemodialysis for an average of 90 months. The mean serum concentration of galectin-3 was 29.68 ± 9.95 ng/ml, and the median PWV was 8.7 m/s (interquartile range: 7.65, 10.61). The baseline demographic characteristics, hemodialysis data, and serum parameters of the entire population are summarized in the first column of Table 1.

**Table 1:** Baseline characteristics.

**Characteristics**	**Overall (*n* = 284)**	**Group 1^a^ (*n* = 56)**	**Group 2 (*n* = 34)**	**Group 3 (*n* = 98)**	**Group 4 (*n* = 96)**	***P***
Age, years	61 (51.25, 68)	54 (38.25, 62)	58.5 (47, 65.25)	64 (58.75, 71.25)	63 (55.25, 70)	<0.001
Male, n (%)	165 (58.1)	33 (58.93)	19 (55.89)	53 (54.08)	60 (62.5)	0.683
BMI, kg/m^2^	21.81 ± 3.33	21.37 ± 3.35	22.93 ± 3.54	21.63 ± 3.17	21.84 ± 3.36	0.164
**Hemodialysis data**						
Dry weight, kg	59.84 ± 11.33	58.28 ± 10.59	63.59 ± 12.55	59.13 ± 11.52	60.13 ± 10.96	0.155
Dialysis duration, months	90 (56, 155)	99 (59, 181)	91 (47.25, 138.25)	82 (48.75, 145.5)	92.5 (62, 158.25)	0.129
Brachial SBP, mmHg	150.46 ± 25.64	136.96 ± 22.55	130.71 ± 20.19	158.76 ± 22.13	156.87 ± 25.75	<0.001
Brachial DBP, mmHg	82.91 ± 13.64	82.14 ± 15.81	78.06 ± 12.50	84.25 ± 12.83	83.70 ± 13.26	0.125
PP, mmHg	46.5 (36.25, 59)	38.5 (30.25, 49.5)	37 (30.75, 41.25)	50 (44, 67)	53.5 (39, 65.75)	<0.001
MAP, mmHg	105.38 ± 16.57	100.59 ± 18.07	95.59 ± 15.38	109.10 ± 14.62	107.84 ± 16.14	<0.001
spKt/V for urea	1.60 (1.41, 1.91)	1.55 (1.41, 2.00)	1.52 (1.33, 1.80)	1.67 (1.44, 1.89)	1.61 (1.43, 1.93)	0.414
**Comorbidities**						
Hypertension, n (%)	238 (83.80)	40 (71.43)	28 (82.35)	90 (91.84)	80 (83.33)	0.011
Diabetes, n (%)	36 (12.68)	0 (0)	3 (8.82)	12 (12.24)	21 (21.88)	0.001
Prior CAD, n (%)	19 (6.69)	3 (5.36)	0 (0)	8 (8.16)	8 (8.33)	0.340
Stroke history, n (%)	19 (6.69)	1 (1.79)	6 (17.65)	6 (6.12)	6 (6.25)	0.032
**Current medication**						
Antihypertensives, n (%)	204 (71.83)	35 (62.5)	22 (64.71)	79 (80.61)	68 (70.83)	0.070
Phosphate binders, n (%)	223 (78.52)	44 (78.57)	32 (94.12)	77 (78.57)	70 (72.92)	0.082
Statins, n (%)	46 (16.2)	8 (14.29)	5 (14.71)	14 (14.29)	19 (19.79)	0.709
**Lab result**						
Total triglyceride, mmol/L	1.46 (1.07, 2.29)	1.43 (0.93, 1.86)	1.91 (1.26, 3.01)	1.58 (1.16, 2.37)	1.37 (1.02, 2.06)	0.051
Total lipoprotein, mmol/L	3.95 (3.34, 4, 59)	3.97 (3.54, 4.41)	4.13 (3.56, 4.46)	3.97 (3.28, 4.90)	3.81 (3.30, 4.53)	0.519
HDL, mmol/L	1.06 ± 0.31	1.11 ± 0.29	1.10 ± 0.38	1.06 ± 0.30	1.01 ± 0.30	0.252
LDL, mmol/L	2.23 ± 0.77	2.16 ± 0.62	2.33 ± 0.73	2.40 ± 0.79	2.05 ± 0.81	0.011
BNP, pg/ml	251.5 (131, 484.75)	276 (103, 553)	210 (121.75, 382.75)	247.5 (150.5, 462.75)	274 (141, 498.5)	0.736
Albumin, g/L	39.33 ± 3.32	39.97 ± 3.42	39.53 ± 3.31	38.74 ± 3.43	39.50 ± 3.08	0.141
CRP, mg/L	2.2 (0.96, 6.15)	1.52 (0.89, 4.11)	1.80 (0.70, 5.89)	2.17 (0.95, 6.57)	3.42 (1.17, 7.37)	0.064
Calcium, mmol/L	2.35 ± 0.26	2.32 ± 0.24	2.37 ± 0.22	2.33 ± 0.29	2.39 ± 0.23	0.335
Phosphorus, mmol/L	1.81 ± 0.51	1.93 ± 0.56	1.84 ± 0.53	1.78 ± 0.44	1.74 ± 0.54	0.170
Creatinine, μmol/L	1,034.35 ± 235.75	1,049.94 ± 238.68	1,116.14 ± 229.72	1,006.05 ± 231.48	1,025.20 ± 236.96	0.117
PWV, m/s	8.70 (7.65, 10.61)	7.11 (6.50, 7.59)	7.15 (6.46, 7.67)	9.72 (8.84, 10.97)	9.69 (8.63, 11.63)	<0.001
Galectin-3, ng/ml	29.68 ± 9.95	21.56 ± 6.03	36.22 ± 3.68	22.90 ± 5.93	39.02 ± 6.61	<0.001

*BMI, body mass index; BNP, B-type brain natriuretic peptide; CAD, coronary artery disease; CRP, C-reactive protein; DBP, diastolic blood pressure; HDL, high-density lipoprotein; LDL, low-density lipoprotein; MAP, mean arterial pressure; PP, pulse pressure; PWV, pulse wave velocity; SBP, systolic blood pressure; spKt/V, single-pool Kt/V*.

a *Group 1: low PWV–low galectin-3; group 2: low PWV–high galectin-3; group 3: high PWV–low galectin-3; and group 4: high PWV–high galectin-3 values*.

### Receiver Operating Characteristic Curve Analysis and Risk Stratification

In the ROC curve analysis (Supplementary Figure 1), the optimal cutoff point for galectin-3 was 30.5 ng/ml (AUC = 0.61, *p* = 0.01), and the cutoff for PWV was 7.9 m/s (AUC = 0.60, *p* = 0.02).

Based on the ROC-optimal cutoffs for PWV and galectin-3, we divided the study patients into four groups: group 1: low PWV–low galectin-3; group 2: low PWV–high galectin-3; group 3: high PWV–low galectin-3; and group 4: high PWV–high galectin-3. The baseline characteristics of these four groups are presented in the second to fifth columns of Table 1. The differences in age, history of hypertension and diabetes, several hemodialysis-related variables, and the low-density lipoprotein level were statistically significant between the four groups.

### Follow-Up and the Primary Endpoint

The median follow-up duration was 31 months. A total of 57 patients (20.1%) reached the composite endpoint of all-cause mortality and MACCE. Twenty-four (57.1%) of the deaths were caused by cerebrovascular or cardiovascular diseases (2 MIs, 3 heart failure cases, 5 sudden cardiac deaths, 10 cerebral hemorrhages, and 4 ischemic strokes), and seven (16.7%) were attributable to infection.

### Univariable and Multivariable Cox Regression Analysis

**Figure 2:**
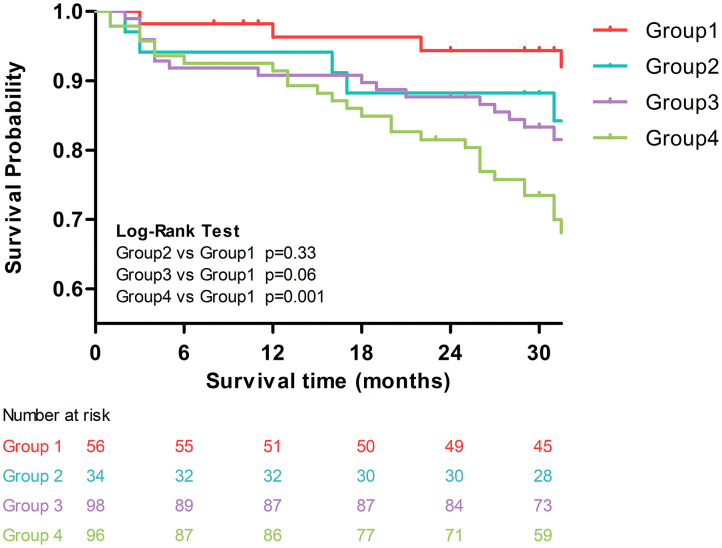
Kaplan–Meier curves of the four groups: group 1: low PWV–low galectin-3; group 2: low PWV–high galectin-3; group 3: high PWV–low galectin-3; and group 4: high PWV–high galectin-3.

In the univariable Cox regression analysis (Supplementary Figure 2), both PWV and galectin-3 were associated with increased risk of the composite endpoint (HR for PWV = 1.11, 95% CI: 1.01–1.22, *p* = 0.033; HR for galectin-3 = 1.03, 95% CI: 1.01–1.06, *p* = 0.014). In addition, age, hypertension, and phosphorus levels were also significant predictors for the composite endpoint. Using group 1 (low PWV–low galectin-3) as the reference group, we found that only group 4 (high PWV–high galectin-3) was associated with a significantly increased risk of the composite endpoint (HR = 4.74, 95% CI = 1.67–13.47, *p* = 0.003). The increased risks in the other two groups were not statistically significant. The Kaplan–Meier curves of these four groups are plotted in Figure 2. Similarly, compared with group 1, group 4 had significantly worse outcomes (i.e., increased risk of composite endpoints, log-rank *p* = 0.001).

In the multivariable Cox regression analysis adjusted for age, mean arterial pressure, albumin, CRP, and phosphorus (Table 2), we observed that only group 4 (high PWV–high galectin-3) was associated with a significantly increased risk of the composite endpoint (HR = 3.19, 95% CI = 1.05–9.66, *p* = 0.04).

**Table 2:** Multivariable Cox regression model for the composite endpoint.

**Variables**	**HR**	**95% CI**	***p-*value**
Age, years	1.04	1.01–1.07	0.011
MAP, mmHg	0.99	0.97–1.01	0.164
Albumin, g/L	0.99	0.90–1.09	0.858
CRP, mg/L	1.01	0.99–1.04	0.245
Phosphorus, mmol/L	0.79	0.43–1.46	0.446
**PWV and Galectin-3 groups^a^**			
Group 1	1 (reference)		
Group 2	1.37	0.34–5.48	0.660
Group 3	1.94	0.62–6.12	0.258
Group 4	3.19	1.05–9.66	0.040

*CI, confidence interval; CRP, C-reactive protein; HR, hazard ratio; MAP, mean arterial pressure; PWV, pulse wave velocity*.

a *Group 1: low PWV–low galectin-3; group 2: low PWV–high galectin-3; group 3: high PWV–low galectin-3; and group 4: high PWV–high galectin-3 values*.

### Model Performance Comparison

We built four multivariable Cox regression models and compared their model performances. Model 1 only included age, mean arterial pressure, albumin, CRP, and phosphorus; model 2 included all variables in model 1 plus PWV; model 3 included all variables in model 1 plus galectin-3; model 4 included all variables in model 1 plus both PWV and galectin-3. Among these four models, model 4 had the best performance in terms of discrimination, calibration, and reclassification (Table 3).

**Table 3:** Model performance comparison.

	**Model 1^a^**	**Model 2**	**Model 3**	**Model 4**
**Discrimination**				
C statistics (95% CI)	0.364 (0.196, 0.601)	0.421 (0.242, 0.523)	0.526 (0.341, 0.622)	0.625 (0.59, 0.66)
**Calibration**				
H-L	*X^2^* = 12.623, *p* = 0.5947	*X^2^* = 9.156, *p* = 0.609	*X^2^* = 7.263, *p* = 0.764	*X^2^* = 4.539, *p* = 0.806
**Reclassification**				
IDI, 95%CI	Reference	0.005 (−0.012, 0.051)	0.006 (−0.009, 0.058)	0.090 (0.049, 0.133)
NRI, 95%CI	Reference	0.102 (−0.167, 0.521)	0.201 (−0.148, 0.407)	0.457 (0.323, 0.573)

*CI, confidence interval; H-L, Hosmer–Lemeshow statistic; NRI, net reclassification improvement; IDI, integrated discrimination improvement*.

a *Model 1 included the following variables: age, mean arterial pressure, albumin, C-reactive protein, and phosphorus; Model 2 = Model 1 + pulse wave velocity; Model 3 = Model 1 + galectin-3; Model 4 = Model 1 + pulse wave velocity + galectin-3*.

## Discussion

Our study innovatively combined PWV with galectin-3 to predict mortality and MACCE in hemodialysis patients. We successfully identified the appropriate cutoffs to dichotomize both measures separately. We demonstrated that patients with high PWV and high galactin-3 levels are associated with a 3-fold increased risk of mortality and MACCE. Our study provides a foundation for incorporating these two measures into the cerebral and cardiovascular risk assessment for hemodialysis patients.

Arterial stiffness is associated with primary coronary events, fatal stroke, and all-cause and cardiovascular mortality in patients with hypertension (13–15), and its prognostic value for mortality has also been confirmed in type 2 diabetes and glucose intolerance patients (16). The association between arterial stiffness and the composite endpoint of MACCE in patients with ST-elevation MI has also been established (17).

PWV is considered the gold standard for measuring arterial stiffness (3, 18). However, it remains controversial whether PWV can predict cardiovascular events in dialysis patients. Blacher et al. first demonstrated that PWV was associated with both all-cause mortality and cardiovascular mortality in ESRD patients. In their multivariable model, each PWV increase of 1 m/s was associated with a 1.39-fold increased risk of all-cause death (19). In the CORD study, Verbeke et al. also confirmed that central arterial stiffness is an independent predictor of mortality and non-fatal cardiovascular events in dialysis patients (4). However, a more recent study showed that the prognostic power of PWV is inferior to that of simple clinical risk scores, and the risk discrimination and reclassification in patients with ESRD was only modestly improved (5). In a head-to-head comparison, the impact of PWV was insignificant when the ankle-brachial BP index was included as a covariate in the multivariable analysis (20). Additionally, the prognostic value of PWV was found to be compromised in elderly patients (6) and dialysis patients with moderate to severe aortic calcification (4).

As a member of the β-galactoside-binding lectin family, galectin-3 can amplify inflammatory and fibrotic processes (21). It has been well-established that a high concentration of galectin-3 is independently associated with all-cause and cardiovascular mortality and an increased risk of heart failure in the general population (22). Galectin-3 has also been shown to be an excellent predictor of mortality in heart failure patients (23). Furthermore, one experimental study demonstrated that inhibition of galectin-3 reduces atherosclerosis in apolipoprotein E-deficient mice (24), suggesting that galectin-3 plays a critical role in the development of atherosclerosis. The association between galectin-3 and atherosclerosis has also been verified by studies in MI patients and patients who received coronary angiography (7, 25).

However, the association between galectin-3 and cardiovascular diseases in CKD patients is unclear. The prognostic power of galectin-3 was found to be compromised by adding renal function into the models (26). Additionally, Zamora et al. found that renal function significantly influenced the prognostic value of galectin-3 in heart failure patients (27). In contrast, in the LURIC study, which was based on a group of patients with impaired renal function, the galectin-3 concentration was significantly associated with all-cause and cardiovascular mortality (10). Consistent with the LURIC study, Obakata et al. found that galectin-3 had independent and incremental prognostic value for all-cause death and a composite of all-cause mortality and MACCE in chronic hemodialysis patients (28).

Although the evidence is conflicting, PWV and galectin-3 are still considered new predictors of cardiovascular diseases. Combining these two indicators may be a reasonable and promising approach for predicting cerebral and cardiovascular risks. Our study showed that appropriate risk stratification could help identify the patients at the highest risk, who will also be candidates for aggressive intervention. There is compelling evidence that combining multiple biomarkers is beneficial for therapy and may improve early risk stratification. In a model in which the risk factors were adjusted, the highest risks of death due to all-causes and cardiovascular death in patients with heart failure were observed when both B-type brain natriuretic peptide and galectin-3 were elevated (29). It is also worth noting that in our study, the model performance comparison showed that adding PWV and galectin-3 can improve model discrimination, calibration, and reclassification.

This study has several limitations. First, it was a single-center observational study that cannot define the cause–effect relationship. Second, all enrolled patients in the current study were Chinese, and the results may not be generalizable to other populations. Third, we did not perform serial measurements of galectin-3 during the follow-up, making it impossible to conduct a longitudinal analysis. Fourth, the sample size in the current study was still limited, and only midterm follow-up results were available. Further studies with a larger sample size and longer follow-up time are warranted.

## Data Availability Statement

The raw data supporting the conclusions of this article will be made available by the authors, without undue reservation.

## Ethics Statement

The studies involving human participants were reviewed and approved by The Ethics Committee of Renji Hospital, Shanghai Jiaotong University. The patients/participants provided their written informed consent to participate in this study. Written informed consent was obtained from the individual(s) for the publication of any potentially identifiable images or data included in this article.

## Author Contributions

QZ and ZN conceived and designed the study. QZ performed the experiments. QZ, MZ, XL, YF, JL, and ZL collected the clinical data. QZ and KY analyzed the data and wrote the paper. ZN reviewed and edited the manuscript. All authors read and approved the manuscript.

## Conflict of Interest

The authors declare that the research was conducted in the absence of any commercial or financial relationships that could be construed as a potential conflict of interest.
