# Supplementary material for: Combining Pulse Wave Velocity With Galectin-3 to Predict Mortality and Cerebrovascular and Cardiovascular Events in Hemodialysis Patients
Source: Front Med (Lausanne). 2020 Oct 20;7:579021. doi: 10.3389/fmed.2020.579021 (PMC7606274; doi:10.3389/fmed.2020.579021)
Supplement: Supplementary file 1 [file Data_Sheet_1.doc]

**Supplementary materials – *Zhang et al.***

**Combining Pulse Wave Velocity with Galectin-3 to Predict Mortality and Cerebrovascular and Cardiovascular Events in Hemodialysis Patients**

**Figure S1: The ROC curve analysis of galectin-3 and PWV as predictors for death and MACCE**

**Figure S2: Univariable Cox regression for composite endpoint**

**Figure S1: The ROC curve analysis of galectin-3 and PWV as predictors for death and MACCE**

**
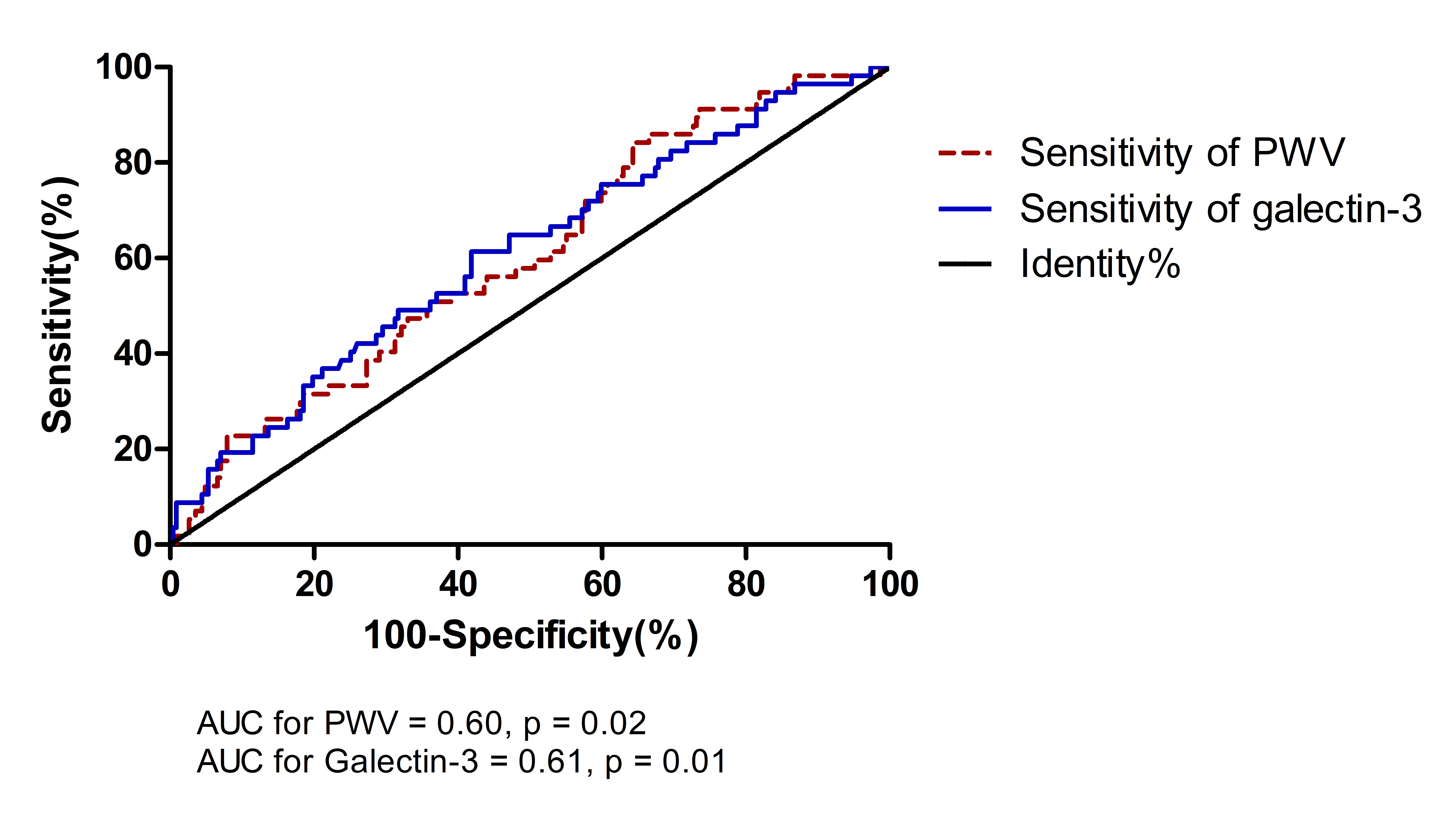
**

**Figure S2: Univariable Cox regression for composite endpoint**
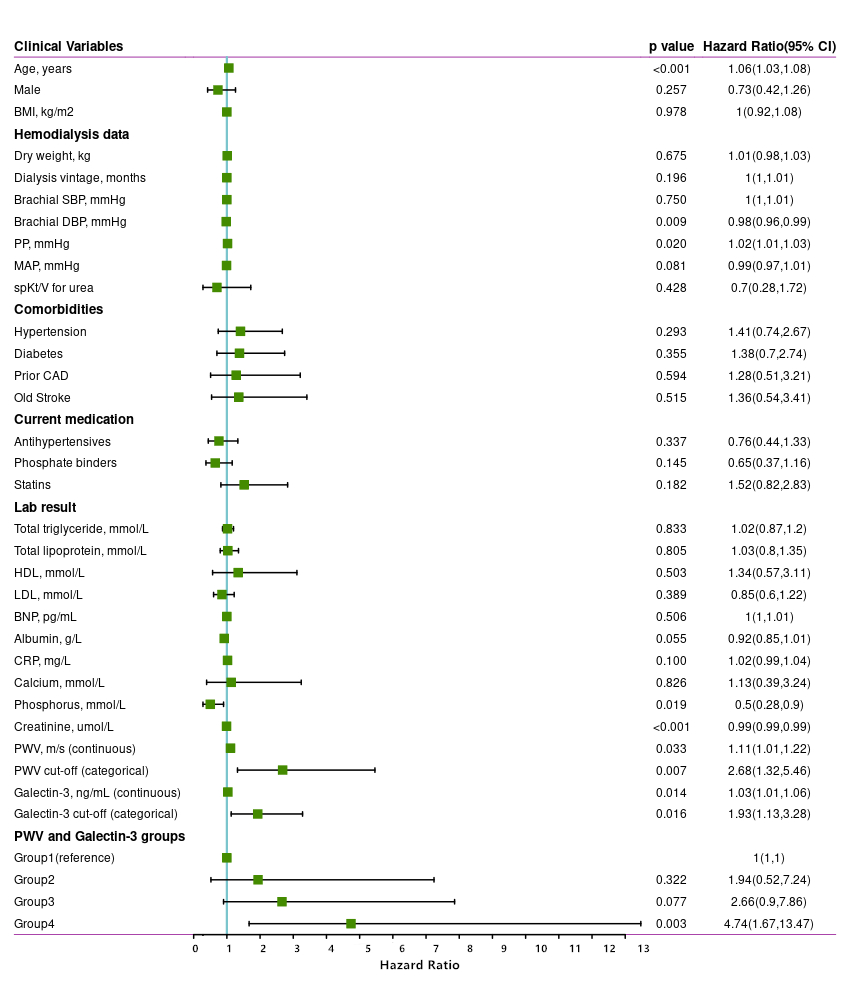
Abbreviations: BMI, body mass index; BNP, B-type brain natriuretic peptide; CAD, coronary artery disease; CI, confidence interval; CRP, C-reactive protein; DBP, diastolic blood pressure; HDL, high-density lipoprotein; HR, hazard ratio; LDL, low-density lipoprotein; MAP, mean arterail pressure; PP, pulse pressure; PWV, pulse wave velocity; SBP, systolic blood pressure; spKt/V, single-pool Kt/V

^a^ Group 1: low PWV - low galectin-3; group 2: low PWV - high galectin-3; group 3: high PWV - low galectin-3; and group 4: high PWV - high galectin-3 values.
